# Reported Events Associated With Spine Robots: An Analysis of the Food and Drug Administration’s Manufacturer and User Facility Device Experience Database

**DOI:** 10.1177/21925682221126116

**Published:** 2022-09-08

**Authors:** Gregory J. Kirchner, Andrew H. Kim, Ariel H. Kwart, John B. Weddle, Jesse E. Bible

**Affiliations:** 1 Department of Orthopaedics & Rehabilitation, 12311 Penn State Milton S. Hershey Medical Center, Hershey, PA, USA

**Keywords:** Robot-assisted spine surgery, screw accuracy, safety, complications, mazor, rosa, excelsius, pulse

## Abstract

**Study Design:**

Cross-Sectional Analysis

**Objectives:**

To summarize medical device reports (MDRs) between August 1, 2017 and November 30, 2021 relating to robot-assisted spine systems within the Manufacturer and User Facility Device Experience (MAUDE) database maintained by The Food and Drug Administration (FDA).

**Methods:**

The MAUDE database was abstract for all MDRs relating to each FDA-approved robot-assisted spine system. Event descriptions were reviewed and characterized into specific event types. Outcome measures include specific robot-assisted spine systems and reported events as detailed by the MDRs. All data is de-identified and in compliance with the Health Insurance Portability and Accountability Act (HIPAA)

**Results:**

There were 263 MDRs consisting of 265 reported events. Misplaced screws represented 61.5% (n = 163) of reported events. Of the 163 reported events, 57.1% (n = 93) described greater than 1 misplaced screw, 15.3% (n = 25) required return to the operating room, 8.6% (n = 14) resulted in neurologic injury, 4.3% (n = 7) resulted in dural tear, and 1.2% (n = 2) resulted in hemorrhage or bleeding. Reported events other than misplaced screws included system imprecision detected prior to screw placement (58/265, 21.9%), mechanical failure (23/265, 8.7%), and software failure (18/265, 6.8%).

**Conclusions:**

As more robot-assisted spine systems gain FDA approval and the adoption of these systems continues to grow, documenting and understanding the range of reported events associated with each “tool” is imperative to balancing patient safety with surgical innovation. This study of the MAUDE database provides a unique summary of reported events associated with robot-assisted spine systems that is not directly linked to a research setting.

## Introduction

Robot-assisted spine systems are increasingly adopted to assist with pedicle screw placement.^1^ Evidence continues to argue more accurate pedicle screw placement with robot-assistance compared to the freehand technique.^1-6^ Furthermore, studies have reported that robot-assisted pedicle screw placement has a similar patient safety profile, reduces radiation exposure, and provides welcomed guidance in cases of complex spinal deformity compared to the freehand technique.^2,7,8^ Nevertheless, robot-assisted spine systems remain a developing technology. Three robot-assisted spine systems were approved by the United States Food & Drug Administration (FDA) prior to the year 2021, while 5 additional systems gained FDA approval during the year 2021.^9,10^

The FDA maintains the Manufacturer and User Facility Device Experience (MAUDE) database, which is an open-access collection of medical device reports (MDRs) pertaining to death, injury, or device malfunction that could potentially result in injury or death relating to any medical device used in the United States.^11^ MDRs are available within the MAUDE database dating as early as 1993, and provide the general public, device manufacturers, patient providers, and health care facilities with timely descriptions of reported events related to a device. Robot-assisted spine systems are included within the MAUDE database. However, to our knowledge, a formal assessment of the robot-assisted spine systems data within the MAUDE database has not been completed.

While the majority of the current data on robot-assisted spine systems is provided by literature on systems used at academic institutions of high-volume centers for spine surgery,^1^ the MAUDE database offers an unbiased collection of reported events relating to these systems. Therefore, the purpose of this study was to provide a succinct summary of the recent data available within the MAUDE database regarding robot-assisted spine systems. This information is imperative for manufacturers, surgeons, and health care facilities in the pursuit of understanding and improving this new “tool” within the field of spine surgery. Furthermore, summaries such as this inform the general public and providers on the range of reported events associated with emerging technology.

## Methods

The MAUDE database is a publicly accessible database of medical device reports (MDRs) maintained by the FDA.^11^ An MDR contains a description of an incident where a device may have caused or contributed to a patient death, injury, or the device malfunctioned in a manner that could potentially lead to death or injury if the malfunction were to recur. MDRs are submitted by mandatory reporters, which include manufacturers, importers and device user facilities, as well as by voluntary reporters such as health care professionals, patients and consumers. The MAUDE database contains data dating back to 1993, and all data is de-identified and in compliance with the Health Insurance Portability and Accountability Act (HIPAA).^11^

All spine robots that were FDA-approved at the time of the study (December 2021) were included. The following robots therefore qualified for inclusion: Mazor X and Mazor X Stealth Edition (Mazor Robotics Ltd., Casesarea, Israel and Medtronic, Minneapolis, Minnesota; FDA approved 2004), ExcelsiusGPS (Globus Medical Inc., Audubon, Pennsylvania; FDA approved 2017), Rosa and Rosa One (Zimmer Biomet Robotics, Montpellier, France; FDA approved 2016), Pulse (NuVasive, San Diego, California; FDA approved 2021), Cirq and Loop X Mobile (Brainlab, Feldkirchen, Germany; FDA approved 2021), Cuvis-spine (Curexo, Inc., Seoul, Korea; FDA approved 2021), and Fusion Robotics (Fusion Robotics, LLC, Boulder, Colorado; FDA approved 2021).^9,10^

For our study, the MAUDE database and its associated “Alternative Summary Reports” were queried using both the “Brand Name” and “Product Code” functions of the online search tool. The following terms were independently queried with the “Brand Name” function: “Mazor,” “ROSA,” “ROSA ONE,” “Excelsius,” “Pulse,” “Cirq,” “Cuvis,” “Loop,” and “Fusion.” Each of these terms was paired with the “Product Code” OLO, which specifies orthopaedic stereotaxic equipment. The terms were also searched using the “Product Code” for neurological stereotaxic equipment (HAW), but none of the results using this “Product Code” described spine systems and instead described systems used for surgery of the brain. A time window was specified between August 1, 2017 and November 30, 2021. The starting point of this time window was chosen based on the date that the third manufacturer of a robot-assisted spine system gained FDA approval, with the intention of limiting the proportion of reported events that may be attributed to the earlier robot systems.

Initial query yielded 281 reported events. Of note, no reported events were found within MAUDE’s associated “Alternative Summary Reports.” Each event description was reviewed, and an MDR was eliminated if it provided insufficient or duplicate information, or information not pertaining to a robot used for spine surgery. A total of 263 complications remained following elimination. Of note, no MDRs were found when querying for “Cirq,” “Cuvis,” “Loop,” or “Fusion.”

Event descriptions were then abstracted for event type. Eleven event types were identified: mechanical failure, software failure, packaging error, imprecision detected prior to screw placement, imprecision detected prior to cage placement, imprecise drilling resulting in hemorrhage/bleeding, misplaced screw(s) without consequence (includes pedicle screw(s) placed not according to plan that were not corrected or corrected intraoperatively), misplaced screw(s) resulting in dural tear, misplaced screw(s) resulting in neurological injury, misplaced screw(s) leading to hemorrhage/bleeding, and misplaced screw(s) requiring return to the operating room. For MDRs involving misplaced screw(s), involvement of greater than 1 screw was recorded.

### Statistical Analysis

Reported events were reported in number and by percentage of total events. Comparative statistics were not performed due to the heterogeneity of reporting with the MAUDE database, as well as the inability to calculate rates of reported events per number of cases.

## Results

### Total Reported Events

A total of 265 reported events were identified among 263 MDRs (Table 1). Of the 265 reported events, 61.5% (n=163) noted misplaced pedicle screws. Involvement of greater than 1 misplaced screw was reported in 57.1% (93/163) of events (Table 2). Of the misplaced screws, 70.6% (113/163) were corrected intraoperatively or not at all, but 15.3% (25/163) of misplaced screws resulted in return to the operating room, 8.6% (14/163) resulted in neurologic injury, 4.3% (7/163) resulted in dural tear, and 1.2% (2/163) resulted in hemorrhage or bleeding. MDRs described imprecision detected prior to pedicle screw placement in 21.9% (58/265) of events (Table 1). Mechanical failure was reported in 8.7% (23/265) of events, while software failure was reported in 6.8% (18/265) of events.

**Table 1.** Reported Events Associated With Spine Robots Within the MAUDE Database

Complication type	Number of Events	Percentage of Events
Misplaced screw(s)	161	61.5%
Imprecise drilling resulting in hemorrhage/bleeding	1	.4%
Imprecision detected prior to screw placement	57	21.8%
Imprecision detected prior to cage placement	1	.4%
Mechanical failure	23	8.8%
Software failure	18	6.9%
Packaging error	1	.4%

**Table 2.** Characteristics Associated With Misplaced Pedicle Screws (n = 163)

Misplaced screw(s)	Number of Events	Percentage of Events
Not corrected or corrected intraoperatively	115	70.6%
Requiring return to OR	25	15.3%
Resulting in neurological injury	14	8.6%
Resulting in dural tear	7	4.3%
Resulting in hemorrhage/bleeding	2	1.2%
Involving >1 screw	93	57.1%

### Mazor X

Of the 265 reported events, 40.8% (n=108) involved the Mazor X robot (Table 3). The most common complication types were misplaced screw without consequence (53/108, 49.1%), imprecision detected prior to screw placement (28/108, 25.9%), and misplaced screw(s) resulting in neurologic injury (9/108, 8.3%).

**Table 3.** Most Common Reported Events Associated With the Mazor X Spine Robot (n = 108)

Complication type	Number of Events	Percentage of Events
Misplaced screw(s) without consequence	53	49.1%
Imprecision detected prior to screw placement	28	25.9%
Misplaced screw(s) resulting in neurological injury	9	8.3%

### Mazor X Stealth

Of the total reported events, 39.2% (104/265) involved the Mazor X Stealth robot (Table 4). The most common complication types were misplaced screw without consequence (56/104, 53.8%), imprecision detected prior to screw placement (25/104, 24.0%), and misplaced screw(s) requiring return to the operating room (15/104, 14.4%).

**Table 4.** Most Common Reported Events Associated With the Mazor X Stealth Spine Robot (n = 104)

Complication type	Number of Events	Percentage of Events
Misplaced screw(s) without consequence	56	53.8%
Imprecision detected prior to screw placement	25	24.0%
Misplaced screw(s) requiring return to OR	15	14.4%

### ROSA ONE

Of the total reported events, 12.1% (32/265) involved the ROSA ONE robot (Table 5). The most common complication types were mechanical failure (14/32, 43.8%), software failure (11, 34.4%), and imprecision detected prior to screw placement (4/32, 12.5%).

**Table 5.** Most Common Reported Events Associated With the ROSA ONE Spine Robot (n = 32)

Complication type	Number of Events	Percentage of Events
Mechanical Failure	14	43.8%
Software Failure	11	34.4%
Imprecision detected prior to screw placement	4	12.5%

### ROSA

Five (5/265, 1.9%) reported events were reported with the ROSA robot (Table 6). These included software failure (3/5, 60%) and mechanical failure (2/5, 40%).

**Table 6.** Most Common Reported Events Associated With the ROSA Spine Robot (n = 5)

Complication type	Number of Events	Percentage of Events
Mechanical Failure	2	40%
Software Failure	3	60%

### Excelsius GPS

Of the total reported events, 4.9% (13/265) were reported with the Excelsius GPS robot (Table 7). The most common complications included misplaced screw(s) requiring a return to the operating room (6/13, 46.2%), misplaced screw(s) without consequence (2/13, 15.4%), and misplaced screw(s) resulting in dural tear (2/13, 15.4%).

**Table 7.** Most Common Reported Events Associated With the Excelsius GPS Spine Robot (n = 13)

Complication type	Number of Events	Percentage of Events
Misplaced screw(s) requiring return to OR	6	46.2%
Misplaced screw(s) without consequence	2	15.4%
Misplaced screw(s) resulting in dural tear	2	15.4%

### Pulse

Three (3/265, 1.1%) reported events were reported with the Pulse robot (Table 8). These included misplaced screw(s) without consequence (2/3, 66.7%) and imprecision detected prior to screw placement (1/3, 33.3%).

**Table 8.** Most Common Reported Events Associated With the Pulse Spine Robot (n = 3)

Complication type	Number of Events	Percentage of Events
Misplaced screw(s) without consequence	2	66.7%
Imprecision detected prior to screw placement	1	33.3%

## Discussion

Recent literature argues more accurate pedicle screw placement with robot-assisted spine systems compared to the freehand technique.^1,6^ However, the source of this evidence is primarily high-volume spine centers or from industry-funded studies.^6,12^ The MAUDE database represents an opportunity to examine reported events associated with the robot-assisted spine systems independent of a research setting. This study of the MAUDE database demonstrates that over half of all MDRs over the preceding 3 years were related to misplaced pedicle screws, with nearly one-third of misplaced screws resulting in a return to the OR or a significant intraoperative complication such as neurological injury, dural tear, or hemorrhage.

There are several notable limitations to this study. Foremost, while reporting to the MAUDE database is mandated by the FDA, the FDA considers the MAUDE database a “passive surveillance system,” whereby the data remains reliant on manufacturers, surgeons, or health care facilities to interpret incidents and to report them to the FDA.^11^ For example, while 1 set of observers might interpret an unplanned reboot of the robot system as a reportable incident, it is possible that another set may not. For this reason, the FDA explicitly states that “the incidence or prevalence of an event cannot be determined” from the MAUDE database.^11^ Therefore, the number of reports in the current study attributed to specific robot systems must be interpreted with caution given that this is likely a consequence of market share and not necessarily a result of higher rates of reported events. Similarly, the level of detail within the event descriptions is dependent on the individual submitting the MDR. Due to insufficient information, a small number of MDRs needed to be excluded from our study. Furthermore, reporting was highly variable on whether misplaced screws were revised intraoperatively with robot navigation or converted to free-hand technique, which is why this information is not specifically reported in our study.

Unsurprisingly, the Mazor and Mazor X Stealth robot-assisted spine systems represented the majority of the MDRs included in this study. In 2004, the FDA approved the first robot-assisted spine system, SpineAssist, manufactured by Mazor Robtics Ltd. (Casesarea, Israel).^13^ Mazor Robotics Ltd. was the sole proprietor of robot-assisted spine systems until the ROSA SPINE (Zimmer Biomet Robotics) was FDA-approved in 2016.^13^ The market share of robot-assisted spine systems remains poorly defined, but the available literature provides a proxy for the adoption of specific systems. For example, a 2017 systematic review by Joseph and Smith^12^ included 25 studies of robot spine systems, which included 24 studies of a Mazor-produced system and 1 study of the ROSA system. A more recent systematic review of randomized controlled trials published in 2021 by Tarawneh and colleagues^6^ included 7 studies, which included 5 studies of Mazor-produced systems and 2 systems that do not have FDA approval. While a significant proportion of studies on robot-assisted spine systems have been conducted outside of the United States, the abundant data on the Mazor systems certainly reflects the predominant share that Mazor robots currently holds within the robot spine system market.

Enhancing the accuracy of pedicle screws is the primary goal of robot-assisted spine systems. A recent meta-analysis of randomized controlled trials comparing robot-assisted and freehand pedicle screw techniques found superior accuracy of robot-assistance with only 31 of 1140 (2.7%) pedicle screws in suboptimal position, as defined as a Gertzbein-Robbins grade of C, D, or E.^6^ While the rate of pedicle screw malposition cannot be determined with the MAUDE database, our finding that 48 misplaced screws resulted in return to the OR, neurological injury, dural tear, or hemorrhage is itself a notable finding. These complications of screw malpositioning are rare within the robot-assisted spine system literature. A study by Kantelhardt et al. recorded reported events, with 7 events (4.7%) in total consisting of 6 dural tears and 1 major hemorrhage.^2^ Reports of return to the OR are limited. Most current literature reports revision rates in terms of conversion to a free-hand technique,^12^ or does not differentiate between conversion and delayed revision.^6^ In a large study of 298 total pedicle screws, Ringel et al. noted only 1 instance of return to the OR for a misplaced screw.^14^

When studying evolving and emerging surgical technologies, it is important to consider the learning curve. The learning curve of using robot-assisted spine systems has been comprehensively studied, including time per pedicle screw, accuracy of screw placement, and fluoroscopy time per screw.^3,14-16^ Evidence suggests that there is a learning curve of approximately 25 to 30 cases before such metrics reach a plateau.^15,16^ However, the robot-assisted spine systems already available to surgeons continue to evolve, and several more systems have been recently introduced in the United States.^9^ This complicates the concept of the learning curve in that the proficiency plateau is routinely disrupted. Considering this, adverse events might be more likely to occur during the learning curve, and providing surgeons with an understanding of those possible events with research such as ours is a valuable opportunity for prevention.

As more robot-assisted spine systems gain FDA-approval and the adoption of these systems continues to grow, documenting and understanding the range of reported events associated with each system is imperative to balancing patient safety with surgical innovation. In this study, the FDA’s MAUDE database provided a profile of reported events incurred in everyday utilization of the robot-assisted spine systems that is not represented within the current literature. While the MAUDE database does not permit calculating the incidence of reported events, our findings identify potential opportunities for enhancing surgical techniques and system designs. As robot-assisted spine systems and their applications continue to evolve, our study underscores the continued need for comparative studies to ensure optimal patient safety profiles.

## References

[1] Li HM Zhang RJ Shen CL . Accuracy of pedicle screw placement and clinical outcomes of robot-assisted technique versus conventional freehand technique in spine surgery from nine randomized controlled trials: A meta-analysis. Spine (Phila Pa 1976). 2020;45(2):E111-E119. doi:10.1097/BRS.0000000000003193 31404053

[2] Kantelhardt SR Martinez R Baerwinkel S Burger R Giese A Rohde V . Perioperative course and accuracy of screw positioning in conventional, open robotic-guided and percutaneous robotic-guided, pedicle screw placement. Eur Spine J. 2011;20(6):860-868. doi:10.1007/s00586-011-1729-2 21384205 PMC3099153

[3] Devito DP Kaplan L Dietl R , et al. Clinical acceptance and accuracy assessment of spinal implants guided with SpineAssist surgical robot: Retrospective study. Spine (Phila Pa 1976). 2010;35(24):2109-2115. doi:10.1097/BRS.0b013e3181d323ab 21079498

[4] Hyun SJ Kim KJ Jahng TA Kim HJ . Minimally invasive robotic versus open fluoroscopic-guided spinal instrumented fusions: A randomized controlled trial. Spine (Phila Pa 1976). 2017;42(6):353-358. doi:10.1097/BRS.0000000000001778 27398897

[5] van Dijk JD van den Ende RP Stramigioli S Köchling M Höss N . Clinical pedicle screw accuracy and deviation from planning in robot-guided spine surgery: robot-guided pedicle screw accuracy. Spine (Phila Pa 1976). 2015;40(17):E986-E991. doi:10.1097/BRS.0000000000000960 25943084

[6] Tarawneh AM Salem KM . A systematic review and meta-analysis of randomized controlled trials comparing the accuracy and clinical outcome of pedicle screw placement using robot-assisted technology and conventional freehand technique. Global Spine J. 2021;11(4):575-586. doi:10.1177/2192568220927713 32677515 PMC8119930

[7] Lieber AM Kirchner GJ Kerbel YE Khalsa AS . Robotic-assisted pedicle screw placement fails to reduce overall postoperative complications in fusion surgery. Spine J. 2019;19(2):212-217. doi:10.1016/j.spinee.2018.07.004 30010044

[8] Urakov TM Chang KH Burks SS Wang MY . Initial academic experience and learning curve with robotic spine instrumentation. Neurosurg Focus. 2017;42(5):E4. doi:10.3171/2017.2.FOCUS175 28463609

[9] Condon A. A breakdown of 7 robots in spine surgery. https://www.beckersspine.com/robotics/item/52042-a-breakdown-of-7-robots-in-spine-surgery.html. Accessed January 12, 2022.

[10] NuVasive gets FDA approval for Pulse platform to assist spine surgeries. https://www.nsmedicaldevices.com/news/nuvasive-pulse-platform-spine-surgeries/. Accessed January 12, 2022.

[11] MAUDE - Manufacturer and User Facility Device Experience. United States Food and Drug Administration. https://www.accessdata.fda.gov/scripts/cdrh/cfdocs/cfmaude/search.cfm

[12] Joseph JR Smith BW Liu X Park P . Current applications of robotics in spine surgery: A systematic review of the literature. Neurosurg Focus. 2017;42(5):E2. doi:10.3171/2017.2.FOCUS16544 28463618

[13] D'Souza M Gendreau J Feng A Kim LH Ho AL Veeravagu A . Robotic-assisted spine surgery: History, efficacy, cost, and future trends. Robot Surg. 2019;6:9-23. doi:10.2147/RSRR.S190720 31807602 PMC6844237

[14] Ringel F Stüer C Reinke A , et al. Accuracy of robot-assisted placement of lumbar and sacral pedicle screws: a prospective randomized comparison to conventional freehand screw implantation. Spine (Phila Pa 1976). 2012;37(8):E496-501. doi:10.1097/BRS.0b013e31824b7767 22310097

[15] Hu X Lieberman IH . What is the learning curve for robotic-assisted pedicle screw placement in spine surgery? Clin Orthop Relat Res. 2014;472(6):1839-1844. doi:10.1007/s11999-013-3291-1 24048889 PMC4016454

[16] Schatlo B Martinez R Alaid A , et al. Unskilled unawareness and the learning curve in robotic spine surgery. Acta Neurochir (Wien). 2015;157(10):1819. doi:10.1007/s00701-015-2535-0 26287268

